# A review on impedimetric immunosensors for pathogen and biomarker detection

**DOI:** 10.1007/s00430-020-00668-0

**Published:** 2020-04-03

**Authors:** J. Leva-Bueno, Sally A. Peyman, P. A. Millner

**Affiliations:** 1 School of Biomedical Sciences, Faculty of Biological Sciences, University of Leeds, Leeds, LS2 9JT England, UK; 2 Molecular and Nanoscale Physics Group, Department of Physics and Astronomy, University of Leeds, Leeds, LS2 9JS England, UK

**Keywords:** Biosensor, Electrochemical impedance spectroscopy (EIS), Immunosensor, Bacteria, Virus, Biomarker

## Abstract

Since the discovery of antibiotics in the first quarter of the twentieth century, their use has been the principal approach to treat bacterial infection. Modernized medicine such as cancer therapy, organ transplantation or advanced major surgeries require effective antibiotics to manage bacterial infections. However, the irresponsible use of antibiotics along with the lack of development has led to the emergence of antimicrobial resistance which is considered a serious global threat due to the rise of multidrug-resistant bacteria (Wang et al. in Antibiotic resistance: a rundown of a global crisis, pp. 1645–1658, 2018). Currently employed diagnostics techniques are microscopy, colony counting, ELISA, PCR, RT-PCR, surface-enhanced Raman scattering and others. These techniques provide satisfactory selectivity and sensitivity (Joung et al. in Sens Actuators B Chem 161:824–831, 2012). Nevertheless, they demand specialized personnel and expensive and sophisticated machinery which can be labour-intensive and time-consuming, (Malvano et al. in Sensors (Switzerland) 18:1–11, 2018; Mantzila et al. in Anal Chem 80:1169–1175, 2008). To get around these problems, new technologies such as biosensing and lab-on-a-chip devices have emerged in the last two decades. Impedimetric immunosensors function by applying electrochemical impedance spectroscopy to a biosensor platform using antibodies or other affinity proteins such as Affimers (Tiede et al. in Elife 6(c):1–35, 2017) or other binding proteins (Weiss et al. in Electrochim Acta 50:4248–4256, 2005) as bioreceptors, which provide excellent sensitivity and selectivity. Pre-enrichment steps are not required and this allows miniaturization and low-cost. In this review different types of impedimetric immunosensors are reported according to the type of electrode and their base layer materials, either self-assembled monolayers or polymeric layers, composition and functionalization for different types of bacteria, viruses, fungi and disease biomarkers. Additionally, novel protein scaffolds, both antibody derived and non-antibody derived, used to specifically target the analyte are considered.

## Introduction

Several worldwide leading healthcare organizations such as the Centers for Disease Control and Prevention (CDC), Infectious Diseases Society of America and the WHO have asserted that antibiotic resistance is a global public threat. Evolution of pathogens along with human beings is a burden and resistance to antibiotics and antivirals are becoming widespread. Several studies reveal that around the 30–50% of antibiotic treatments are inappropriate in the USA, where antibiotic-resistant hospital acquired infections (HAIs) are responsible for 99,000 deaths annually [1]. According to O’Neill’s report within the UK, the problem of AMR by 2050 is estimated to put in risk 10 million human lives per year and will have spent a cumulative amount of 100 trillion USD [7]. Therefore, early detection of these microorganisms in humans or animals is essential to provide efficient and adequate treatment.

Currently employed diagnostics techniques are: microscopy, microbial culture, ELISA, PCR, RT-PCR, multiple-tube fermentation (MTF), SERS, and others. Microscopy is a very convenient technique to assess morphological features, but is less sensitive than microbial culture, which is particularly time-consuming, often more than 24 h. Besides, microbial culture has low sensitivity and is relatively expensive [8]. Some immunoassay methods such as ELISA can specifically detect epitopes on bacterial surfaces. However, diagnostics based on ELISA are time-consuming, expensive, have a complex and narrow detection range and often show cross-reactivity [9]. PCR is appropriate for tiny samples and results in highly specific detection. However, PCR lacks reliability due to frequent false positive outcomes [10]. MTF consists of filtering a water sample, concentrating the bacterial cells and incubating them for later detection and quantification. Since the technique depends on bacterial growth, one test can last up to 96 h [11]. Finally, the SERS technique requires expensive and sophisticated laser equipment [12].

To overcome the preceding methodological difficulties, new technologies such as biosensing have emerged in the last few decades. This technology can detect and quantify biological analytes and combine high sensitivity and specificity with fast response times, portability, low-cost, and ease-of-use.

## Biosensors

**Fig. 1:**
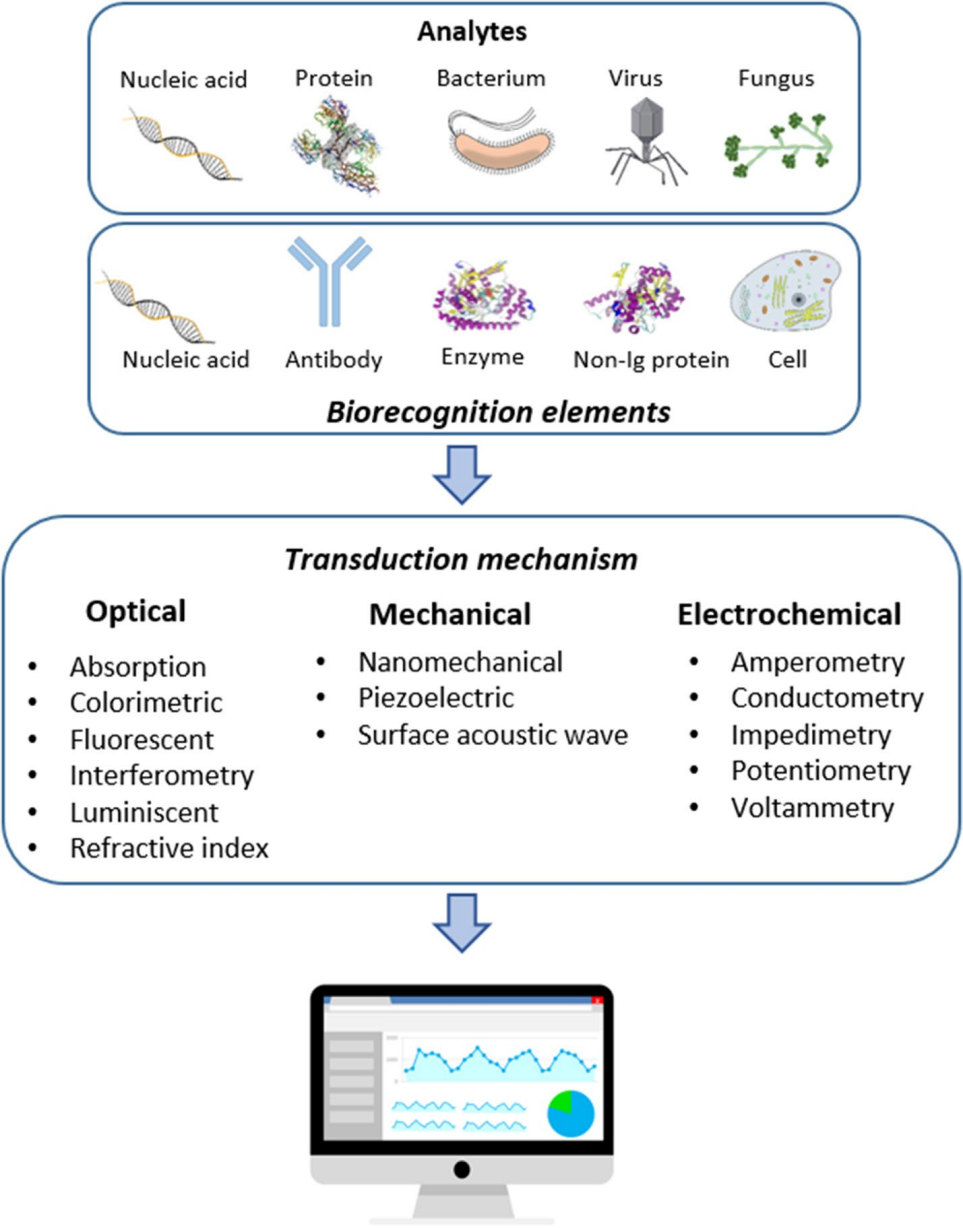
Schematics of biosensor platforms: general overview of biosensors in which different types of biorecognition elements and transduction mechanisms are shown and a signal processor. Some vectors are reproduced from CSIRO ScienceImage

A biosensor [13] is defined as a compact analytical device that detects and quantifies a target analyte and consists of three elements: a biological receptor (DNA, antibodies, enzymes, cells) which specifically detect the target molecule; a transducer, which interprets the biological recognition event and translates it into a quantifiable signal; and a signal processing display. The rise of biosensors is due to the limitations that current techniques present such as high costs, requirement for qualified personnel and long response time. All of these complications are incompatible with early-stage rapid diagnosis (Fig. 1).

The first biosensor [14] dates back to 1962. An electrochemical biosensor was developed to measure glucose levels in blood. The enzyme glucose oxidase (GOx) catalyses the oxidation of the glucose, and the biosensor monitored its consumption through the change in the current at the working electrode. Since then, electrochemical biosensors have drawn the attention of several research areas due to their easy manipulation, great sensitivity and possibility for miniaturisation.

Biosensors for medical application are designed to detect and quantify biomedical analytes. Pathogens found in different bio-fluids such as saliva, urine and blood can be detected [15, 16]. Nowadays, implantable biosensors have been already developed and are in use in some patients, for instance for a constant monitoring of glucose [17].

Biosensors can be classified either by their bioreceptor element or by the transduction mechanism. Where antibodies are used as bioreceptors they are called immunosensors. Antibodies are one of the most important bioreceptors to target specific analytes, taking advantage of the highly specific non-covalent interaction between antibodies and antigen [18]. Classifying by the type of transducer, the main categories are: optical, mechanical and electrochemical [19]. This last category, electrochemical, can be subdivided in to amperometric, potentiometric, conductometric and impedimetric [20].

### Optical biosensors

Optical biosensors are based on the measurement of absorbance, reflectance, or fluorescence emission in the UV, visible, or near-infrared (NIR) [21]. The advantages of optical biosensors are mainly due to their high sensitivity, ability to monitor in real-time and the possibility of being label-free. The most utilized label-free transduction method is surface plasmon resonance (SPR).

SPR is a charge-density oscillation that occurs in the interface of the metal and the dielectric. In SPR, a plasmon wave is measured over a metal surface, and works by applying a light through the biological sample, causing a change in the refractive index, which is used to monitor the binding [22]. The commercialization of SPR is already well established. However, optical method can also suffer the disadvantage of interference by coloured analytes and many bio-fluids, for instance blood and urine, which show significant colour. Besides, SPR as well as many other optical methods requires sophisticated and expensive machinery [23, 24].

### Mechanical biosensors

Mechanical biosensors detect changes in the properties of the surface upon a biological binding event. The sensor records surface stress or oscillation frequency due to mass deposition. Different type of mechanical biosensors are: surface-stress mechanical biosensors, that measures the change in deflection of the cantilever when a biomolecule binds to the surface. A laser beam is used to obtain the position of cantilever deflection; dynamic-mode mechanical biosensors, in which the device oscillates at a fixed resonance frequency, that changes upon biomolecule binding. Other types of biosensors are quartz crystal microbalance (QCM) and whispering-gallery microgravity (WGM). Although these techniques are robust, as they monitor surface mass they are less sensitive to small molecules and non-specific deposition can be an issue [25].

### Electrochemical biosensors

Electrochemistry is the branch of the chemistry that interrelates electrical and chemical reactivity [26]. Electrochemical transduction is one of the most abundant modes nowadays due to its rapid response time, user-friendly application, low-cost production and the possibility of miniaturizing the system. The basis of an electrochemical biosensor is to measure the changes that take place in the proximity of the electrode surface. There, electrons flow between the electrode surface and electrolytes in solution. The changes at the electrode surface are monitored through parameters such as electrolyte resistance, charge transfer at the electrode surface or mass transfer from the bulk solution to the electrode surface. For electrochemical biosensors, transduction can be subdivided in the following: potentiometric, amperometric and impedimetric [27].

#### Potentiometric sensors

Potentiometric biosensors [28] measures the potential at the working electrode, at zero current, with respect to the reference electrode. This measurement takes place under equilibrium conditions, when no current is flowing through the electrochemical cell. This technique is used to assess ion concentrations across the electrode surface. These biosensors combine biorecognition elements, mainly enzymes, with a transducer that measures the uptake or release of ions that occurs during the enzyme’s action on its target. Ions such as Na^+^, K^+^, Ca^2+^, H^+^ or NH_4_^+^ can all be measured, but typically are H^+^ or NH_4_^+^. Many potentiometric sensors are essentially modified pH electrodes [29]. The relationship between the free ion concentration and the potential is established by the Nernst equation (Eq. 1) and of course the stoichiometry between ion and target substrate enables calibration.

1 $$E_{{{\text{cell}}}} = E_{{{\text{cell}}}}^{0} - \frac{RT}{{nF}}\ln Q,$$

where *E*_cell_ is the cell potential, $E_{{{\text{cell}}}}^{0}$ is the standard cell potential, *R* is the universal gas constant, *T* the temperature, *n* is the charge number of electrode reaction, *F* is the Faraday constant and *Q* is the ratio of ion concentrations between the anode and the cathode.

In the case of voltammetric biosensors, the current is monitored as a result of the application of a varied potential. The three most known voltammetry techniques are potential step, linear sweep, and cyclic voltammetry (CV). CV is also useful in biosensor fabrication at the step of polymer-layer deposition, known as electropolymerisation [28, 30].

#### Amperometric

Typically, an amperometric biosensor measures the current at a constant potential [31]. The first practical biosensor was the glucose biosensor, which measured the depletion of oxygen and the change in current by the enzyme catalysed reaction of glucose oxidase (GOx) (Eq. 2) [14]. This biosensor was the first of what is called the first generation glucose biosensors, which are characterized for the employment of oxygen as a cosubstrate and the generation and detection of hydrogen peroxide (Eq. 3). The first generation was quickly superseded by replacing the need for oxygen by an electron mediator, such as ferrocene or potassium ferricyanide, which acts to reoxidise the lavin cofactor, leading to the second generation of glucose biosensors. Subsequently, the reduced mediator is oxidized at the electrode surface such in Eqs. 4, (5) and (6) [32]. Later improvements consisted of developing biosensors in which the electron transfer was carried out without the use of electron mediators. Instead, there was a direct exchange of electrons between the enzyme and the electrode. This is called third generation glucose biosensors. However, recent improvements and continuous glucose monitoring are being achieved by non-enzymatic glucose biosensors. These biosensors are considered the fourth generation glucose biosensors and are characterized for using a catalytic electrode for glucose oxidation. These electrodes are modified by electrodeposition, etching or electrochemical anodization [33].

2 $${\text{Glucose } + \text{ Oxygen}}\overset {{\text{GOx}}} \longleftrightarrow {\text{Gluconic acid } + \text{ Hydrogen peroxide}}$$

3 $${\text{H}}_{{2}} {\text{O}}_{{2}} \to {\text{O}}_{{2}} {\text{ } + \text{ 2H}}^{ + } {\text{ } + \text{ 2e}}^{ - }$$

4 $${\text{Glucose } + \text{ GOx}}_{{\text{(ox)}}} \to {\text{Gluconic acid } + \text{ GOx}}_{{\text{(red)}}}$$

5 $${\text{GOx}}_{{\text{(red)}}} {\text{ } + \text{ 2M}}_{{\text{(ox)}}} \to {\text{GOx}}_{{\text{(ox)}}} {\text{ } + \text{ 2M}}_{{\text{(red)}}} {\text{ } + \text{ 2H}}^{ + }$$

6 $${\text{2M}}_{{\text{(red)}}} \to {\text{2M}}_{{\text{(ox)}}} {\text{ } + \text{ 2e}}^{ - }$$

Generally, in amperometric biosensors label-free is practically not possible and often an oxidoreductase enzyme catalyses the biochemical reaction, which turns into a change in current across the electrode surface, proportional to analyte concentration. These biosensors allow rapid responses and high sensitivity. However, their use are limited to analytes of which there are specific enzymes to catalyse the redox reaction. Faraday’s Law (Eq. 7) can determine the relationship:

7 $$I = n \cdot F \cdot A \cdot J,$$

where *I* is the current, *n* the number of electrons transferred to the electrode, *F* the Faraday constant, *A* the area of the electrode and *J* is the Flux coefficient.

Amperometric biosensors show better sensitivity than potentiometric biosensors. Nevertheless, these systems require enzymes to oxidise or reduce a specific analyte [34], which is a limitation on their use. Commercial amperometric biosensors determine the presence and concentration of glucose in animals and microbial cultures [35], lactose and other metabolites [36] and some lipids such as cholesterol [37].

#### Impedimetric

Impedance is basically the opposition to the current flow in an electrical circuit. The difference between common resistance and impedance is that resistance obeys Ohm’s law and occurs in direct current (DC) circuits, where there is no gap between the voltage applied and the current. Impedance, however, occurs in full alternating current (AC) circuits, where there is a gap in the voltage-current phase angle due to appearance of capacitive and inductive effects. In the case of impedimetric biosensors, the impedance consists of a resistive and a capacitive part as a result of a complex interaction with a small amplitude voltage signal as a function of frequency [38, 39].

Unlike amperometric and potentiometric systems, impedance biosensors are label free and do not depend on any specific enzyme for the analyte detection. Instead, impedimetric biosensors rely on a unique bioreceptor which specifically binds to the analyte such as DNA [40], antibodies [41, 42] aptamers [43] and various synthetic affinity proteins such as Affimers [5, 44].

## Brief description of electrochemical impedance spectroscopy (EIS)

### Electrochemistry at electrode surface

**Fig. 2:**
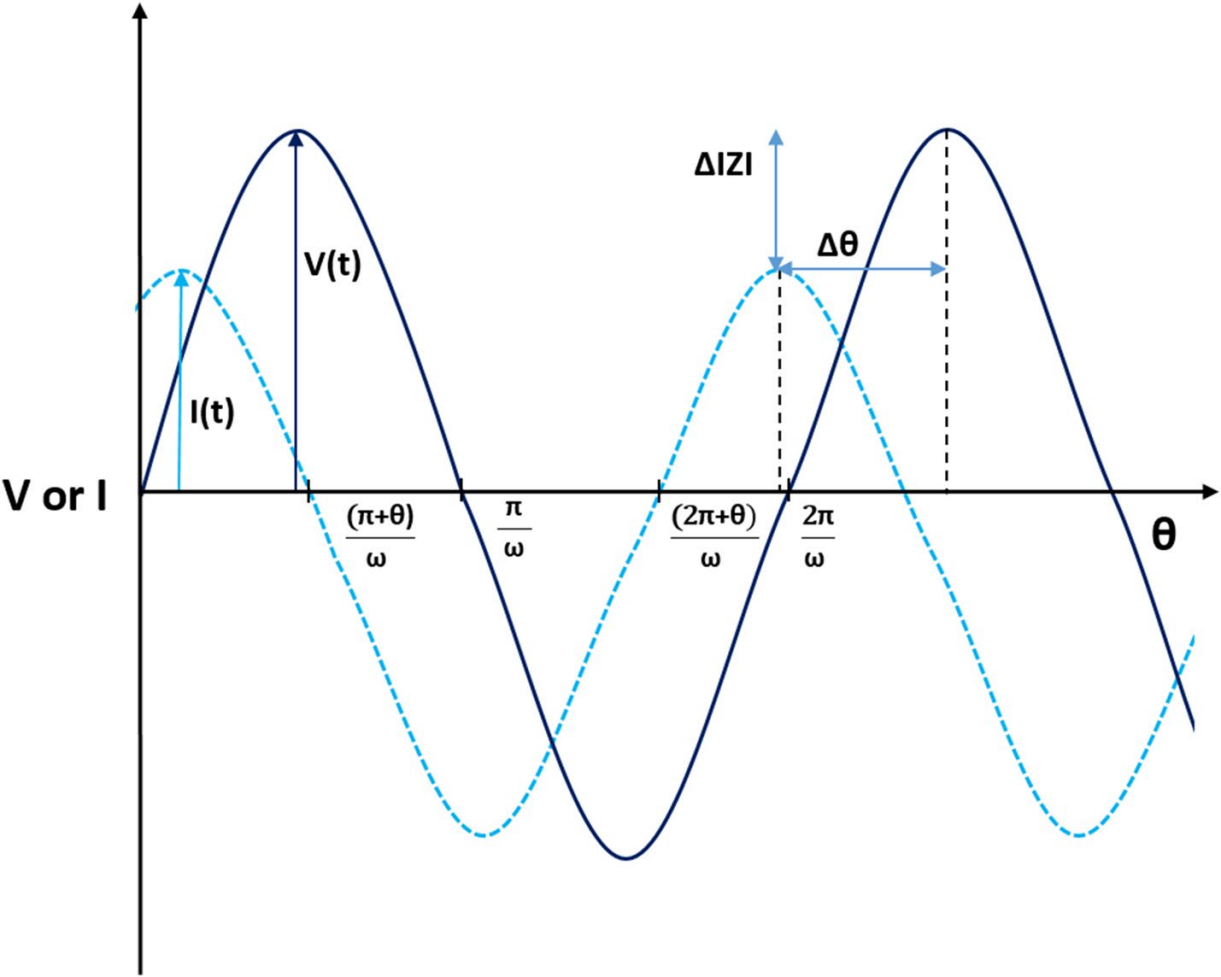
Phasor diagram: a phasor diagram shows the change in phase angle (*θ*) and magnitude (|*Z*|) when analyte binding occurs

When EIS is used on a biosensor, several parameters are obtained. Bulk impedance (*Z*) can be separated into real and imaginary component, which are the resistive (*Z*′) and the capacitive (−*Z*″) parts, respectively (Fig. 2). The resistive part is originated by the electrode surface through the opposition to the current flow. The capacitive part measures the storage of charge of the system when a voltage is applied. At the electrode-solution interface, there are two ways in which electricity can flow through the electrode: when the electrons are transferred to the electrodes by means of redox reactions it is called a Faradaic process and behaves according to Faraday’s law. When no charge is transferred to the electrode surface, however, electricity can flow through since the system behave as a capacitor, it is called a non-Faradaic process [45, 46].

The basis of EIS consists of applying a low amplitude voltage sine wave to an electrochemical system over a range of frequencies. As a result, the current and its phase angle is obtained. The impedance is the ratio between the applied voltage and the current (Eq. 8) and indicates the opposition of an electrical circuit to the flow of electrons in the AC circuit. Impedance-based biosensors culminate with a bioreceptor-analyte interaction which causes modifications to the electrical field due to a change in the capacitance and electron transfer resistance at the working electrode surface [39].

8 $$Z\left( {j\omega } \right) = \frac{{V\left( {j\omega } \right)}}{{I\left( {j\omega } \right)}},$$

where *Z* is the impedance, *V* is the voltage, *I* is the current, *j* is the imaginary component and *ω* is the frequency.

When the phase angle between the voltage and intensity is equal to zero, as in a pure metal surface, the impedance and resistance become the same. Nevertheless, in the majority of real electrical circuits the angle is different from zero as a consequence of capacitive and/or inductive effects.

To assess the impedance of an electrochemical system in Faradaic biosensors, the modified surface of the biosensor electrode is immersed in a solution containing an electron mediator. The most commonly used redox pairs include [Fe(CN_6_)]^3−/4−^ (ferricyanide/ferrocyanide), [Ru(NH_3_)_6_]^3+/2+^ hexaammineruthenium (II/III) and ferrocene (Fc^+^/Fc). In non-Faradaic circuits, impedance is measured without a redox mediator [13].

Usually, a SAM or a polymer layer are used to cover the electrode surface. This coating provides a surface to immobilize the bioreceptors, increases their bio-stability and creates a dielectric between the surface and the media. Self-assembled monolayers (SAMs) require a molecularly flat surface, incompatible with the roughness presented by a gold screen-printed electrode (SPE) surface. Alternatively, conducting and non-conducting polymers are typically electrodeposited onto metal surfaces. These polymers are good base layers since they can be electrodeposited onto rough surfaces and the thickness of the polymer itself can be controlled. Conducting polymers such as polyaniline (PANI) or polypyrrole (PPy) exhibit conductive or semi-conductive properties whereas non-conducting polymers like polytyramine (Ptyr) offer high resistivity and contribute to highly sensitive detection [46, 47].

### Equivalent circuit and data presentation

**Fig. 3:**
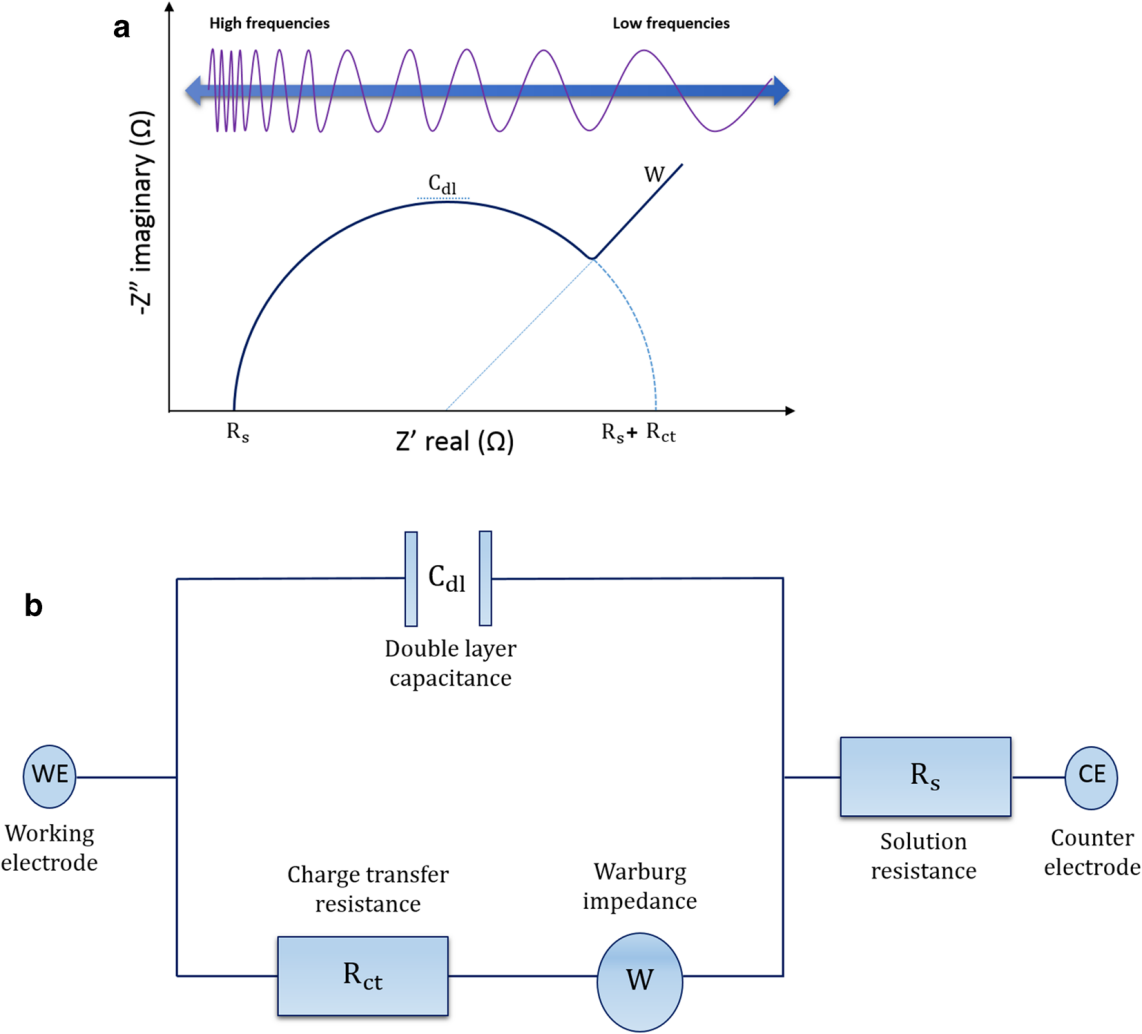
General scheme of a Nyquist plot and its Randles’ equivalent circuit: **a** Nyquist plot emerges from plotting the imaginary (capacitive) against real (resistive) components of impedance. Most relevant features are: resistance of the solution *R*_s_; charge transfer resistance *R*_ct_; the maximum double-layer capacitance *C*_dl_ and the Warburg impedance *W*, which is only observed in Faradaic sensors and represents mass transfer diffusion effects. **b** Randles’ equivalent circuit representing an electrical circuit modelling a Faradaic sensor

Common formats for impedance data presentation are the Nyquist and Bode plots. In the Nyquist plot (Fig. 3a), the imaginary part of impedance (−*Z*″), out of phase, is plotted against the real component (*Z*′), in phase, at each excitation frequency whereas the Bode plot, shows the logarithm of absolute impedance and phase shift (Θ) versus the log of excitation frequency. The Nyquist plots shows the relationship between real and imaginary components of impedance for a range of frequencies and are typically used to assess the resistive component of the system whereas Bode plots are used to study mainly the capacitive [48, 49]. The Nyquist plot is explained in detail for the common use of data assessment in this project (Fig. 3a).

The behaviour of the system is different at high and low frequencies. At high frequencies, the signal is controlled by kinetic processes: electron mediator molecules change in charge direction before the redox reaction at the electrode surface takes place. This fact constitutes a limiting factor since it delays the charge transfer across the electrode. This limitation is known as solution resistance (*R*_s_). At medium frequencies, there is barely resistance in the system. As a consequence, the changes originated in the system are due to capacitance, specifically from the double layer capacitance (*C*_dl_) at the electrode surface. At low frequencies, charge transfer is only produced by the resistance offered from the biosensor construct, since the opposition found by the electron mediators is due to the surface components. This resistance is calculated through the charge transfer resistance (*R*_ct_) [50]. In some occasions, at low frequencies, is seen Warburg impedance (*W*), which is manifested as a linear tail at the end of the Nyquist arc. This phenomena occurs due to diffusional limitations of the systems, when it runs out of charge carriers [46]. The equivalent circuit for all these components is represented on a Randles’ equivalent circuit (Fig. 3b) which models the behaviour of a typical Faradaic biosensor.

**Fig. 4:**
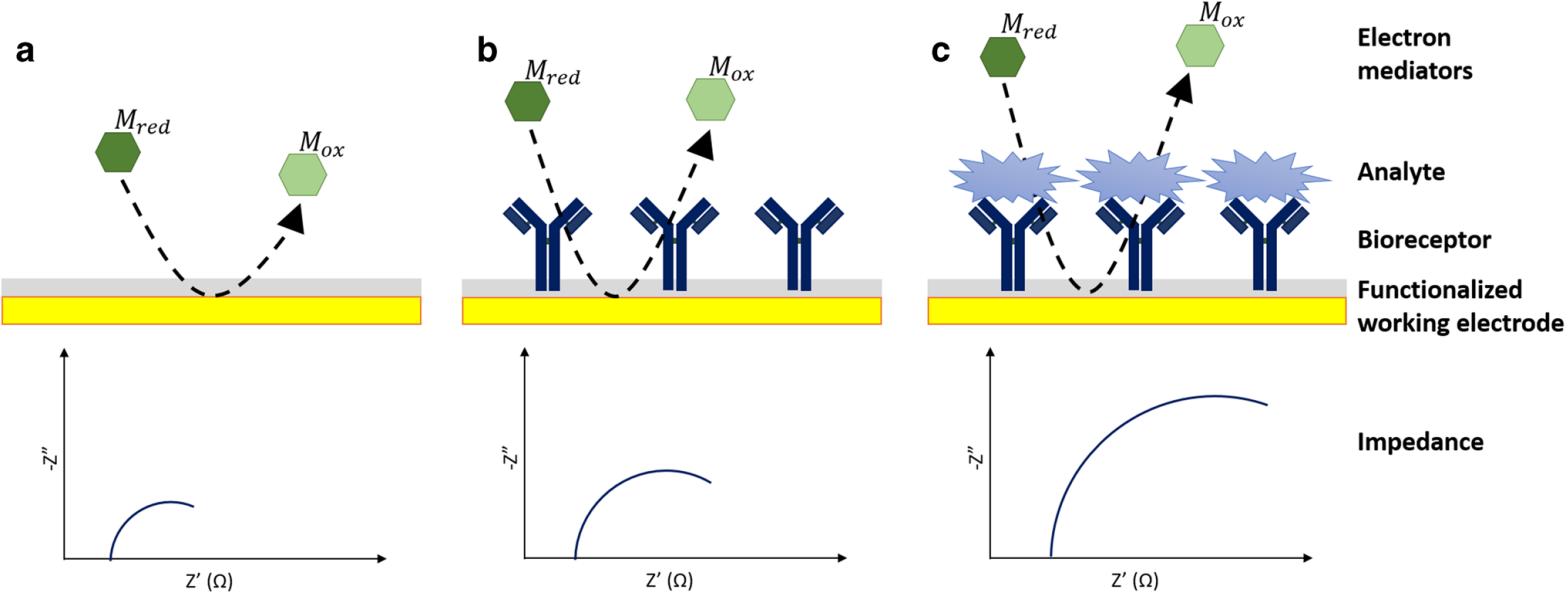
General scheme of impedance for each step of biosensor construction. Impedance increases as the deposition over the surface electrode increases. Deposition of material onto biosensor surface normally causes an increase in both resistance and capacitance, which impedes the transfer of electrons between the solution-based mediators and the electrode surface. Therefore, impedance increases from (**a**), bare electrode when (**b**), bioreceptors are immobilized and (**c**), increases upon analyte binding

Generally, the tendency of the impedance value is to increase as the complexity of the functionalized electrode increases: electrons in solution face more obstacles when reaching the electrode surface and thus, more resistance is observed (Fig. 4). However, a decrease in impedance can occur upon analyte binding, since analyte binding can distort the polymer layer and make electron mediator access easier [51].

In Faradaic measurements, the main aim is to calculate the charge transfer resistance and the current flowing through the biosensor surface. In contrast, in non-Faradaic measurements, a voltage at low frequencies is applied and the current flows through the biosensor after accumulating the capacitive components of the surface. In general terms, Nyquist plots are typically used for resistive systems whereas Bode plots are used for capacitive [48, 49].

In this review the progress of impedimetric immunosensors field in the last two decades is discussed, according to a wide range of pathogens and biomarkers. The characteristics of a biosensor are the working electrode, target element, immobilization step, limit of detection (LOD), detection time and sample volume. Previous reviews about biosensors classify them either by type of bioreceptor or by type of transducer. In this case, the bioreceptor consists of an antibody and the transducer component is an impedance electrode. Thus, the classification in this review is organised by type of analyte: bacteria, virus and fungi, biomarkers and a final section of new protein receptors.

## Immunosensors for pathogen detection

### *Escherichia coli *(*E. coli*)

*Escherichia coli* is a gram-negative bacteria primarily found in the intestines of mammal and birds. Common strains are not harmful even at high concentrations. However, a few *E. coli* strains can cause illness, for instance strain O157:H7 which produces a haemorrhagic toxin and causes haemorrhagic colitis [52]. Impedimetric immunosensor platforms for *E. coli* have been the object of many studies due to its easy manipulation in the laboratory and it being a surrogate organism for sensing faecal pollution of water [53].

One of the first relevant cases of an *E. coli* impedance immunosensor [54] employed gold interdigitated array microelectrodes (IDAMs) and were modified and inserted in a microfluidic device [55] for *E. coli* O157:H7 detection. The system was capable of detecting concentrations as low as 1.2 × 103 CFU/mL from ground beef samples and 1.6 × 102 CFU/mL from pure culture in only 35 min using 100 µL of sample. Specific antibodies against *E. coli* were conjugated to magnetic nanoparticles via biotin-streptavidin coupling, forming magnetic nanoparticle-antibody conjugates (MNAC). These conjugates captured bacteria and concentrated them after applying a magnetic field. Eventually, the sample was placed into the microfluidic device for impedimetric assessment. Unlike macro-sized electrodes, which generally consists of a metal bar immersed into a solution, the use of IDAMs in impedance immunosensors [56] presents several advantages. For instance, lower detection limit, higher signal/noise ratio, shorter detection time and lower sample volume. The IDAMs’ design is based on a pair of parallel microband array electrodes that mesh into each other forming a set of interdigitated electrode fingers. Frequently dimensions used for IDAMs are 0.1–0.2 µm high for each electrode finger, 1–20 mm in length with an inter-electrode space of 1–20 µm. Gold is the most prevalent material for IDAM fabrication. However, the use of indium tin oxide (ITO), Pt, Ti, Pd or Rh is also mentioned [20, 55].

*Escherichia coli* O157:H7 detection in river water [57] was achieved by covering gold electrodes with a mercaptoacetic acid (MACA) SAM. Subsequently, the construct was treated with *N*-ethyl-*N*-(dimethylaminopropyl)-carbodiimide (EDC) and *N*-hydroxysuccinimide (NHS) to catalyse the creation of a peptide bond with antibodies. The co-addition of NHS and EDC causes the replacement of the terminal carboxylic group of MACA by an NHS ester which is then subjected to nucleophilic attack by an amine group. An LOD of 1 × 10^3^ CFU/mL analysing a small sample volume of 20 µL was achieved in 1 h, which compared to 100 µL sample [54] decreases the volume despite longer detection time but was still acceptable. Usually, SAMs forms an insulation barrier between the electrode and the analyte solution, thus behaving as a dielectric. This dielectric barrier is used to investigate electron transfer [58].

**Fig. 5:**
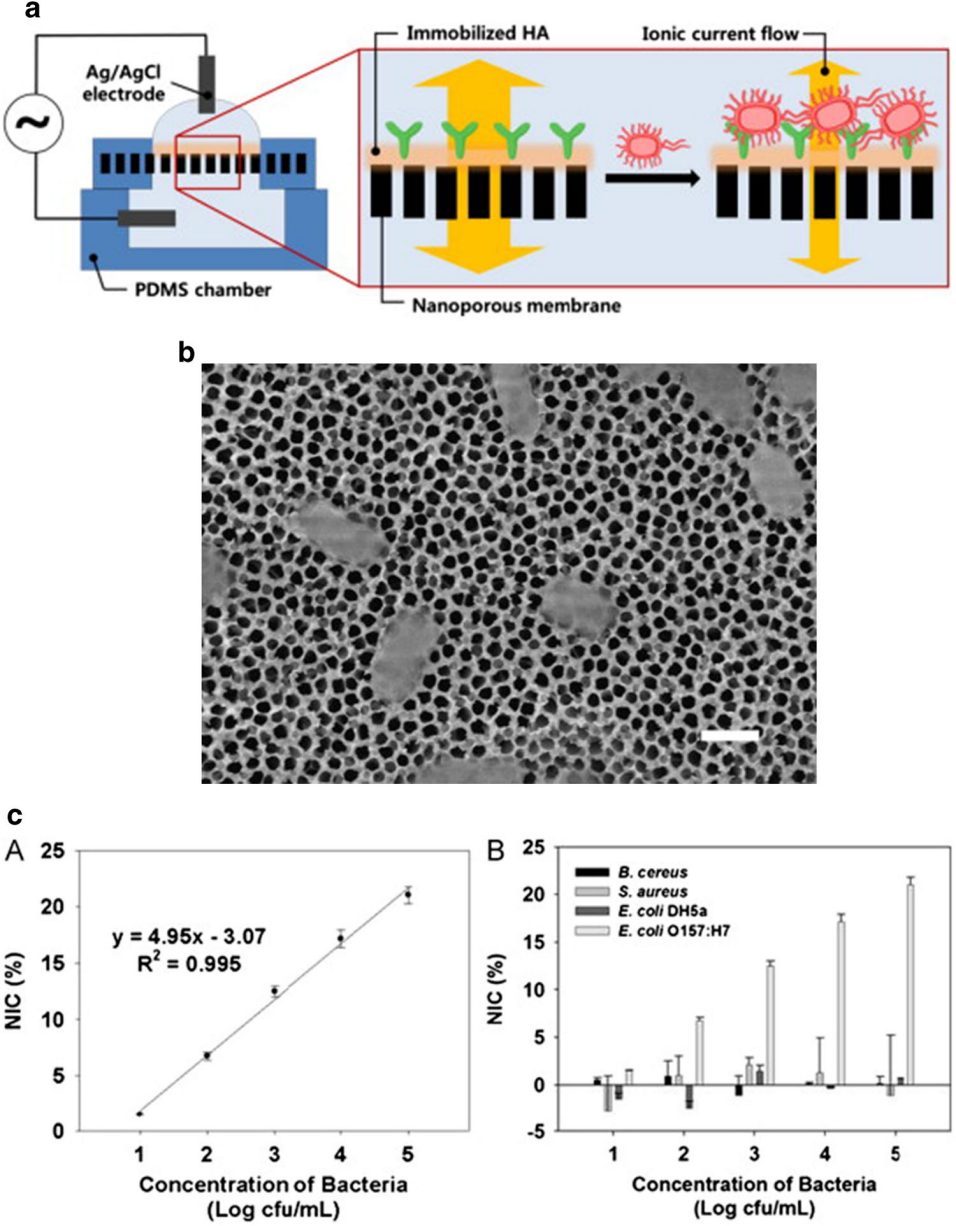
**a** Scheme of the impedimetric immunosensor constructed into a HA-coated alumina nanoporous for *E. coli* detection inserted into a microfluidic device. **b** A FE-SEM image showing bacteria captured over the nanoporous membrane. Scale bar is 1 μm. **c** the plot on the left shows a normalized impedance change (NIC) for different bacteria concentration and the plot on the right shows the validation experiments through negative control bacteria. Reprinted from [61], with permission from Elsevier

A microfluidic biosensor platform was describe for *E. coli* and *Staphylococcus aureus* (*S. aureus*) detection [59]. On this occasion, antibodies were attached onto a modified alumina nanoporous membrane with self-assembled (3-glycidoxypropyl) trimethoxysilane (GPMS) SAM. The use of nanoporous alumina membrane in impedimetric immunosensing is due to the increase in the electron transfer through the electrode-solution interface caused by its high pore density, successful biocompatibility and expansion of surface area. Moreover, these membranes are extraordinarily long-lasting and the pore size can be regulated without difficulty [60]. Both type of bacteria were detected in 2 h and at an LOD of 10^2^ CFU/mL was achieved. Alumina nanoporous membrane was also reported [61] but adding a modification with hyaluronic acid (HA) (Fig. 5). HA is a hydrophilic non-sulphated glycosaminoglycan which is used in immunosensor construction to improve the signal/noise ratio by decreasing non-specific background signals. It also enhances antibody immobilization due to its carboxyl groups. The biosensor platform could detect concentrations of *E. coli* as low as 83.7 CFU/mL in milk samples. Therefore, the introduction of HA improved the previous sensitivity obtained in [59]. In other cases of biosensor fabrication using HA [2], introducing a conducting polyaniline (PANI) film surface prior to antibody immobilization [62] led to a low LOD of 2 CFU/mL [27] being reported.

**Fig. 6:**
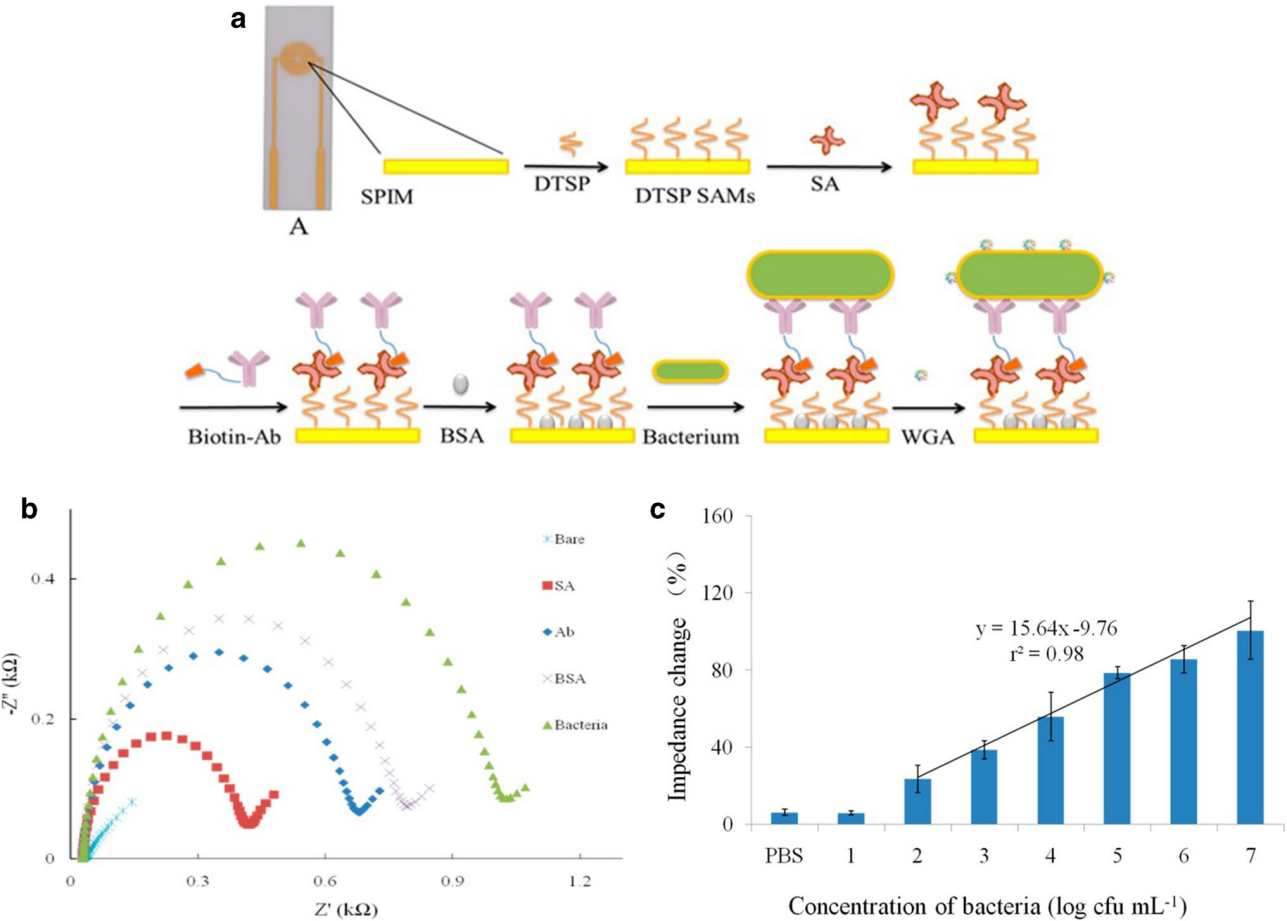
**a** Scheme for layer-by-layer construction of the impedimetric detection immunosensor. **b** Nyquist plot of each step electrode functionalization. For bare electrode the impedance value is almost negligible. As the complexity of the electrode surface increases the impedance also does since the pathway of the electrons to get the surface is more hindered. **c** Impedance change (%) plotted against log (CFU/mL) of bacteria concentration. The increase in bacteria concentration creates a thicker platform, which turns an increase in the impedance value. Reproduced from [66]

Reduced graphene oxide paper (rGOP) electrode modified with gold nanoparticles (AuNPs) was used as a novel system for *E. coli* O157:H7 detection in cucumber and ground beef samples [53]. The AuNPs were electrodeposited onto the graphene paper and antibodies were linked to them via biotin-streptavidin coupling. Graphene, in addition to its advantages of biocompatibility, rapid electron transfer and large specific area surface, is characterised for its high flexibility and thus their great importance in impedimetric immunosensors. Incorporation of AuNPs in the biosensors construction was carried out due to their well-known functionalization chemistry and electrochemical properties [63]. AuNPs create an appropriate microenvironment for the stabilization and immobilization of biomolecules as well as ease the electron transfer between the bulk material on the electrode and the electrode itself [64]. Some impedimetric immunosensors require an amplification step in order to increase sensitivity, for instance by employing AuNPs [65] or the lectin wheat germ agglutinin (WGA) (Fig. 6). In this case, screen-printed interdigitated microelectrodes (SPIMs) was used as a biosensor platform, covered with 3-dithiobis-(sulfosuccinimidyl propionate) (DTSP), and further functionalized until depositing antibodies. Once the bacteria was tested, WGA served as a signal amplifier [66]. The biosensor functionalization can be followed in (Fig. 6a) scheme and each step monitored through impedance and show in Nyquist plot in (Fig. 6b). Different bacterial concentrations are plotted by % change in impedance in (Fig. 6c).

Magnetic nanobeads served as a substrate to be coated with antibodies [67]. These antibody-coated magnetic nanobeads are used for separating and transporting the bacteria from the initial culture into another platform for electrochemical measurement and for concentrating and precipitating the pathogen onto the electrode by placing a magnet under the electrode. Common materials for working electrodes are gold, silver, platinum and carbon. However, a biosensor platform was built over an (ITO) electrode [68] and showed successful results: an LOD of 1 CFU/mL in a 400 µL PBS sample could be obtained in 45 min. Similarly, a gold-tungsten microwire was used as a working electrode modified with polyethyleneimine (PEI) SAM for *E. coli* K12 detection [69]. One of the latest impedimetric immunosensor fabrication [3] tried 5 distinct manners of functionalizing the electrode surface with anti-*E. coli* onto a gold SPE and detected an LOD of 3 CFU/mL in 90 min for a 1 mL sample. Considering main important features such as LOD, detection time and sample volume, the biosensors using ITO electrode [68] and gold SPE [3] provide better sensitivity whereas the biosensors using gold IDAM [54] and gold electrode with a MAA SAM [57] use considerably smaller sample volumes, thus less invasive towards the obtaining sample from patients. All four detection could be carried out in a relative short detection time, from 35 to 90 min.

### *Salmonella*

*Salmonella* is a gram-negative bacteria which belongs to the *Enterobacteriaceae* family. The most prevalent types of *Salmonella* which infect humans are *Salmonella typhimurium* (*S. typhimurium*) and *Salmonella typhi* (*S. typhi*). *S. typhimurium* is the less virulent and its common symptoms appear 12–72 h after infection. They include fever, diarrhoea, abdominal colic and headache. However, *S. tiphi* causes typhoid fever disease, causing around 200,000 deaths per year and a morbidity of approximately 20 million new cases per year [70].

An immunosensor for *S. typhimurium* evaluation in milk samples [71] was fabricated using a gold working electrode covered with a thiol-based SAM in which antibodies against *Salmonella* were attached via glutaraldehyde cross-linking. The immunosensor platform could attain an LOD of 10^2^ CFU/mL in a 2 mL PBS after 2 h whereas an LOD of 10^2^ CFU/mL was obtained after 10 h in a 2 mL milk sample. Similarly, antibodies were also attached to the SAM via glutaraldehyde [72]. One thousand (10^3^) CFU/mL in 1 mL of pathogen could be detected in only 20 min, thus improving results published in [71]. Gold IDAMs were employed for immunosensor construction [73] using novel magnetic silica nanotubes (MSNTs) to capture bacteria through electrostatic interaction. The use of MSNTs is due to their multifunctional structure and for being less susceptible to self-aggregation under elevated levels of salt in the media. Another cases using a gold IDAM [74] was able to detect in 1 h an LOD of 10^2^ CFU/mL in only 50 µL volume, thus improving previous sample volumes reported in [71, 72]. For *S. typhi* detection [75] AuNPs were coated with antibodies and an LOD of 10^2^ CFU/mL in 10 µL sample could be achieved in 1 h.

### Sulphate reducing bacteria (SRB)

**Fig. 7:**
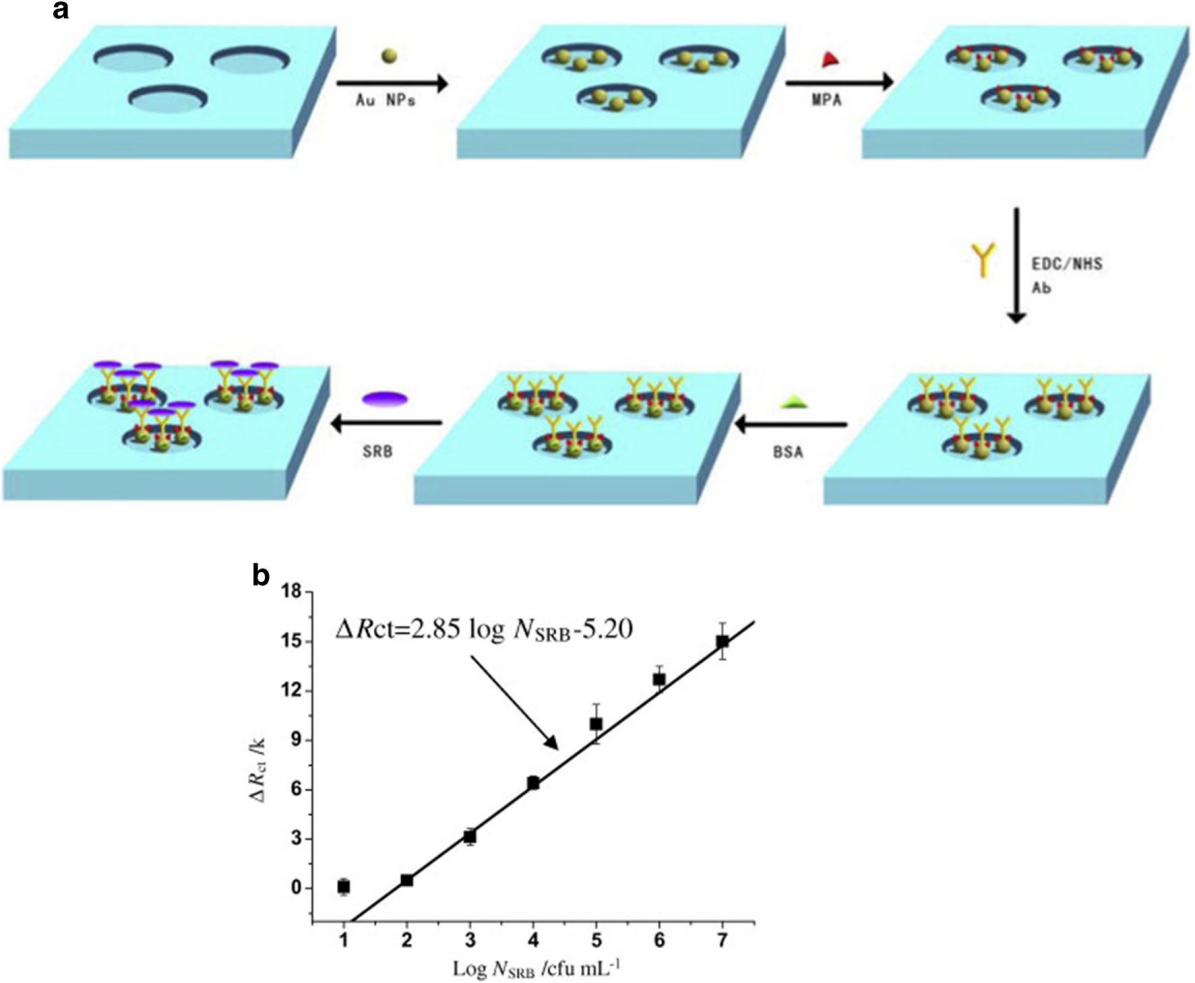
**a** General scheme of the biosensor construction for SRB detection over 3D-foam Ni foam. **b** Change in *R*_ct_ plot showing a calibration curve for different bacterial concentrations. Reprinted from [78], with permission from Elsevier

The electron transport chain of *Desulforibrio caledoiensis,* an SRB, possesses a sulphate as a terminal electron acceptor and thus, produces sulphide, which is known for being a major problem for industries and the environment. One of the first impedimetric immunosensor for SRB detection immobilized the lectin-concanavalin A (ConA) for an agglutination assay [76]. The gold electrode was modified with a SAM onto which the lectin ConA was immobilized. Each lectin molecule presents four carbohydrate-combining sites. Therefore, when lectins react with cells they will also cause cross-linking and then precipitation [77]. A immunosensor for SRB [78] introduced the utilization of a Ni foam, covered with 11-mercaptoundecanoic acid (MPA), which worked as a platform for trapping bacteria. This system is depicted in Fig. 7. Further inclusion of reduced graphene sheets (RGS) in the SRB biosensor [79] was also reported. These 2D nanostructures are biocompatible, provide a redox catalyst and a low manufacturing cost [80]. A RGS-doped chitosan nanocomposite film biosensor platform could detect a range of bacteria at concentrations from 1.8 × 10^2^ to 1.8 × 10^7^ CFU/mL. In comparison to the work on SRB detection [76, 78], this RGS-based immunosensor obtained faster results and, even though the sensitivity was not improved, the sample amount could be as low as 10 µL.

### Listeria monocytogenes

*Listeria monocytogenes* is a gram-positive bacteria whose infection takes place via contaminated food ingestion. Generally, the diseases caused by this bacteria includes febrile gastroenteritis, perinatal infection and systemic infections in which the central nervous system is affected. Hence, faster and cheaper methodologies such as impedimetric immunosensor have been researched [81].

An immunosensor [82] for *Listeria monocytogenes* employed a TiO_2_ nanowire bundle microelectrode. Pathogen detection was accomplished in 50 min for an LOD of 4.7 × 10^2^ CFU/mL in 15 μL sample. This clearly outperformed the common immunoassay detection and dot blot assay, with LODs of 10^4^ CFU/mL and 2.2 × 10^5^ CFU/mL respectively. The use of a TiO_2_ nanowire is mainly due to its unique semi-conductive band gap not found in other nanowires, favourable biocompatibility and good chemical and photochemical stability, as well as easy fabrication [82]. Forward steps towards miniaturisation and low volume samples were also achieved [83] by inserting a biosensor into a micro fluidic device. Magnetic nanoparticles 30 nm diameter were coated with antibodies against the pathogenic bacteria via biotin-streptavidin coupling. The system could analyse bacteria in a 3-h immunoreaction for food samples (milk, ground beef and lettuce), achieving an LOD of 10^4^ CFU/mL. Due to microfluidic chip use, the volume sample required was only 20 nL, which justifies the limitation of sensitivity when using minimal sample volume. The main features of using a microfluidic device for biosensing include an increased ratio surface/volume and the insertion of small volumes in the order of nanoliters inside the microchannels, turning into a considerable reduction of the detection time and minimising the cost of reagents [83].

### *Pseudomonas aeruginosa*, *Streptococcus pyogenes* and *Staphylococcus aureus*

**Fig. 8:**
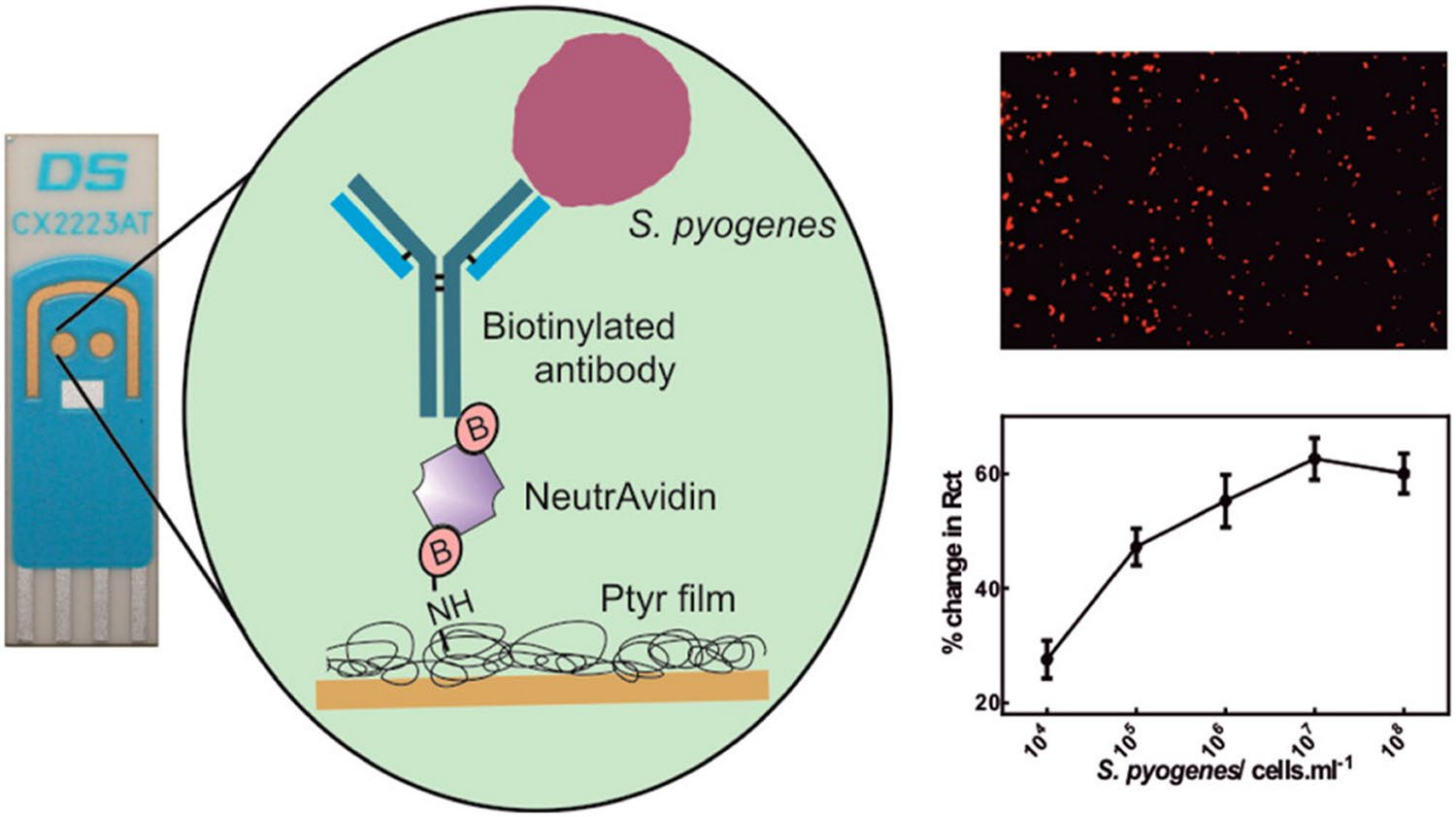
On the left, there is the general scheme of immunosensor against *Streptococcus pyogenes* construction layer-by layer over a DropSens gold SPE (CX2223AT). On the top right, a fluorescence imaging of bound *S. pyogenes* on the sensor surface. On the bottom right, there is a % change in impedance plot upon the addition of different bacteria concentration, from 10^4^ to 10^8^ cells/mL. Reproduced from [41], with permission of ACS

*Pseudomonas aeruginosa* is a gram-negative bacteria commonly found in contaminated water. Infections by this pathogen in humans is manifested as urinary tract infections, respiratory system infections and systemic infections among others. An impedimetric immunosensor for this pathogen [84] was designed by immobilising polyclonal antibodies against *P. aeruginosa* over a screen-printed carbon electrode (SPCE). *Streptococcus pyogenes* is a gram-positive bacterium responsible of pharyngitis, scarlet fever (rash) impetigo, cellulitis, or erysipelas. An immunosensor was described [41] in which Dropsens gold SPEs were modified by depositing a polytyramine (Ptyr) layer. Subsequently biotin tagged antibodies were attached via biotin-NeutrAvidin. An LOD of 10^2^ cells were achieved for single shot incubation method with a sample volume of 10 µL in only 30 min (Fig. 8). *S. aureus* is a gram-positive bacteria member of the *Micrococcaceae* family. Infections caused by this pathogen include diseases such as pneumonia, heart valve infections and bone infections which produces a considerable morbidity and mortality. An impedimetric immunosensor for stressed and resuscitated *S. aureus* assessment [85] was developed by modifying a gold electrode with an insulating 6-mercaptohexadecanoid acid SAM in which specific antibodies against *S. aureus* were attached for the further immunoreaction.

### Viral and fungal detection

Avian Virus Influenza (IV) H5N1 is highly pathogenic and mainly occurs in birds. However, human infection by this virus is generally associated with several disease and death. A biosensor platform for IV H5N1 detection [86] immobilized polyclonal antibodies against IV H5N1 surface antigen HA (hemagglutinin) over a modified gold IDAM via protein A. An LOD of 10^3^ EID_50_/mL (EID_50_: 50% egg Infective Dose) and linear detection range from 10^3^ to 10^7^ CFU/mL was achieved in 2 h in a 50 µL sample. Protein A adsorbs easily onto the gold IDAM surface through electrostatic and hydrophobic interactions. Besides, this protein shows high affinity to the anti-H5N1 IgG Fc region, which facilitates antibody immobilization onto the electrode surface. Similarly, IV H5N1 detection in chicken swabs was achieved [38]. A gold IDAM was used as an electrode platform and was functionalised via protein A with monoclonal antibodies against IV H5N1. Concentrations from 2^–1^ to 2^4^ HAU/50 µL (HAU: hemagglutination units) could be assessed in a 30 µL sample after 45 min.

Other impedimetric immunosensors include a regenerable biosensor for adenovirus type 5 (Ad5) detection [87], a biosensor for plum pox virus (PPV) [88] which affects plants from genus *Prunus* and a biosensor platform for the pathogen oomycete fungus *Aphanomyces invadans* [89], known to cause epizootic ulcerative syndrome (EUS).

### Biomarker detection

#### Biomarkers for cardiovascular disease (CVD)

Cardiovascular diseases (CVD) is a major cause of death worldwide, estimated to be up to 30%. Several disorders such as deep vein thrombosis (DVT), congenital heart disease, pulmonary embolism, cerebrovascular disease, coronary heart disease, rheumatic heart disease and peripheral artery disease are include in CVD. Some unmodifiable risk factors of suffering from a CVD are the gender, age, ethnicity and family history whereas among modifiable risk factors can be found in tobacco use, sedentary lifestyle, hypertension, obesity, hyperlipidemia and stress. According to epidemiological studies, preventive measures are the best treatment for CVD [90]. This fact leads to monitor different biomarkers related with CVD for early diagnostics.

**Fig. 9:**
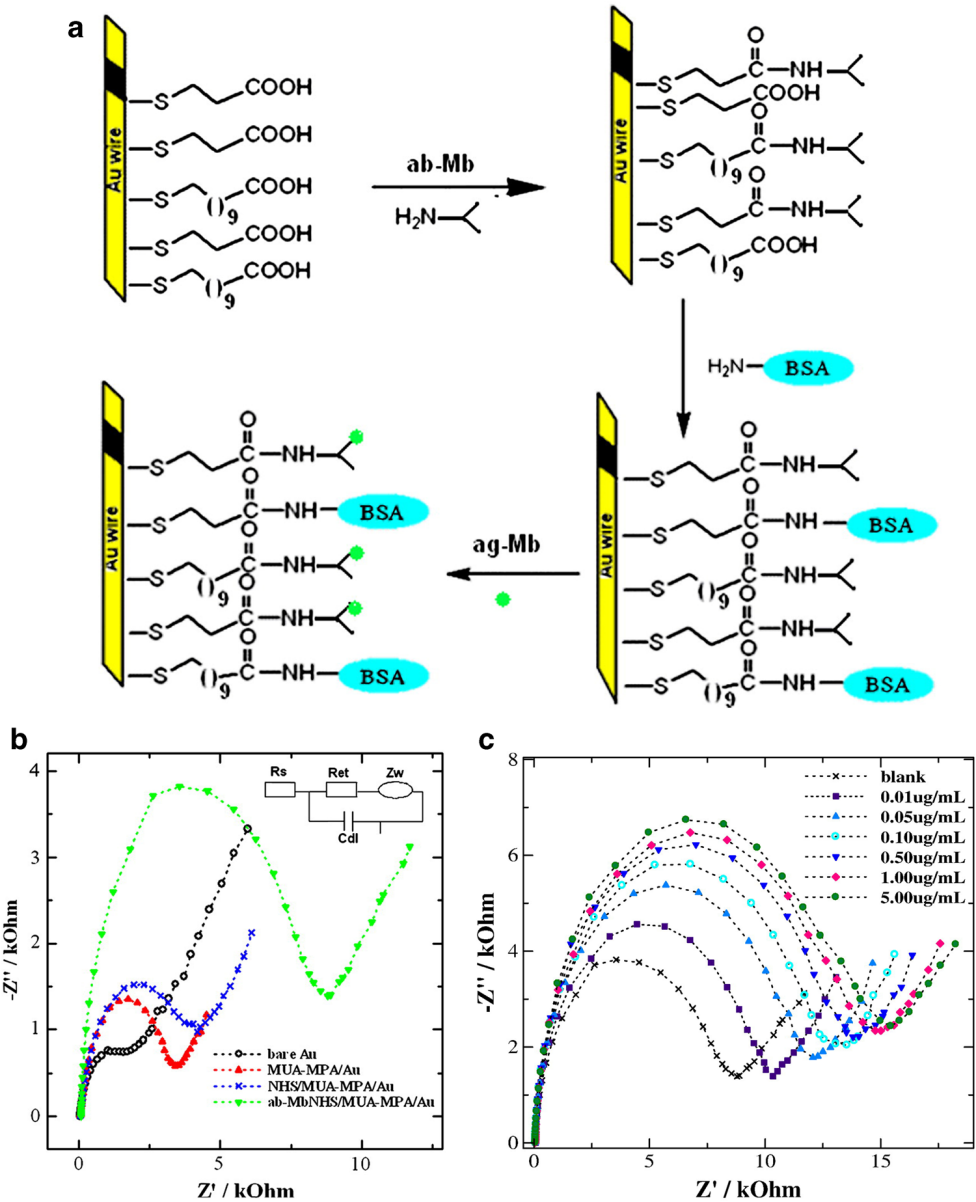
**a** Scheme of the biosensor construction for Mb detection. A flat gold wire is used as electrode to be functionalized with anti-Mb, blocked with BSA, and finally tested with Mb; **b** shows the impedance values for each step of the biosensor construction; and **c** the impedance values shown through a Nyquist plot for increasing concentration of the analyte. Reprinted from [91], with permission from Elsevier

Myocardial infarction (AMI) requires of a rapid and accurate diagnostic. Myoglobin (Mb), cardiac troponins (cTn), creatine kinase MB (CK-MB) and myeloperoxidase (MPO) are indicators of elevated risk of AMI. In 2010, an impedimetric immunosensor for Mb was constructed over a flat gold wire (Fig. 9). Myoglobin, which consists of a 17.8 kDa protein, was detected in aqueous solution over linear range from 10 to 650 ng/mL, with an LOD of 5.2 ng/mL [91]. Further impedimetric immunosensors for Mb have been developed. An impedimetric immunosensor for Mb was achieved by [92], reaching an LOD of 1.70 ng/mL and could detect a linear range from 0.01 to 1 μg/mL in less than 15 min for samples in PBS. Several novel components were included in the biosensor architecture. An indium-tin-oxide (ITO) glass plate was employed as electrode platform, in which a SAM was deposited and then functionalized with platinum nanoparticles prior to antibody attachment. However, the most sensitive impedimetric immunosensor for Mb was achieved by [93], in which Screen-printed multi-walled carbon nanotubes electrodes (MWCNTs) [94] could improve the sensitivity of Mb detection. An LOD of 0.08 ng/mL could be achieved and a linear range from 0.1 ng/mL to 90 ng/mL could be detected in 5 µL samples. Several novel components were included in the biosensor architecture. An indium-tin-oxide (ITO) glass plate was employed as electrode platform, in which a SAM was deposited and then functionalized with platinum nanoparticles prior to antibody attachment. Other CVD biomarkers have also been detected though impedimetric biosensing. Cardiac troponin I (cTnI), which is used as a definitive biomarker for AMI diagnosis and soluble lectin-like oxidized LDL receptor-1 (sLOX-1), which serves as a biomarker for early diagnostic of AMI and acute coronary syndrome (ACS), were detected in PBS and in serum samples. The sensitivity of the final sensor could detect an LOD of 10^–13^ M for each analyte [95]. A more recent investigation, lead to a better biosensor platform for cTnI detection. An LOD of 11.7 fM was achieved and a linear range from 42 fM to 42 nM could be detected in approximately 1 h [96]. The biosensor construction included the addition of dendrimer before antibody attachment, which improved the LOD over 120 times. The reliability of the biosensor prototype was checked in parallel with ELISA, and resulted in a robust method for cTnI detection in serum. Biosensors have also reached other diseases such as deep vein thrombosis (DVT), which is typically indicated by the appearance of D-dimer. A platform based on a gold microelectrode was functionalized with single-walled carbon nanotubes (SWCN) and anti-D-dimer in order to detect the mentioned molecules at levels of 0.1 pg/mL (53 fM) in less than 10 min [97].

#### Biomarkers for cancer

Several types of cancer such as ovarian, breast and pancreatic cancer can be early detected by assessing the levels of the protein human epidermal growth factor receptor (EGFR). A disposable CNT based biosensor was developed and could detect EGFR at low levels such as 2 fg/mL, improving the LOD of 4 pg/mL provided by commercial kits by then [98]. Another biosensor for EFGR detection was developed by depositing AuNPs and LODs of 0.34 pg/mL and 0.88 pg/mL were achieved for samples in PBS and in human plasma respectively [99]. Clinical observational methods have demonstrated that 1 out of 3 men over 50 years old have evidence of histologic prostate cancer, although in a several number of cases the tumour is small and insignificant [100]. Then, it is important to develop rapid detection platforms for early-diagnosis. A gold microelectrode was used a platform to construct an impedimetric immunosensor for prostate-specific antigen (PSA) detection and quantification, which is a biomarker overexpressed in prostate cancer. An LOD in the order of ng/mL was achieved [101].

The progression of ovarian cancer can be followed by monitoring the serum oncomarker cancer antigen 125 (CA-125). For that, an impedimetric immunosensor for CA-125 determination was developed over a gold electrode platform that was previously functionalized with silica coated gold nanoparticles and quantum dots [102, 103]. The system could detect CA-125 in serum of ovarian cancer patients with an LOD of 0.0016 U/mL in less than 1 h [104]. Negative regulator protein murine double minute 2 (MDM2) serves as a tumour brain marker. A biosensor for MDM2 was develop to detect this protein in health mice and mice with brain tumours. For that, a biosensing platform was built over a polycrystalline gold electrode. MDM2 biomarker was detected in PBS and brain homogenate samples, with an LOD of 0.29 pg/mL and 1.3 pg/mL respectively, both in less than 1 h, and thus improving the detection time of commercial kits which last for 5 h [105]. Paediatric adrenocortical carcinoma (pACC) is an unusual cancer typically found in South America. It is characterized for the high production of dehydroepiandrosterone sulphate (DHEAS). This pACC biomarker was detected by developing a biosensor over an oxidised glassy carbon electrode that was functionalized with AuNPs. An LOD of 7.4 μg/dL in blood serum samples was achieved [106].

#### Biomarkers for bacterial and other diseases

Triggering receptor-1 expressed (TREM-1) is a biomarker which indicates a response to bacterial sepsis, whilst *N*-3-oxo-dodecanoyl-l-homoserine lactone (HSL) is present in pathogenic wound infections. Therefore, the need for a rapid detection of these molecules can be crucial for a fast response towards wound infection. The construction of an impedimetric immunosensor for these biomarkers was accomplished [52]. Gold SPEs were modified with antibodies and the detection was achieved in less than 1 h in a 10 µL mock wound samples. LODs were 3.3 pM for TREM-1 and 1.4 nM for HSL, which are near or below the limits required to consider the presence of infection.

Tuberculosis is one of the most prevalent and important worldwide diseases due to its virulence and death rate among the centuries. This disease is caused by *Mycobacterium tuberculosis*, which affects mammals and it is estimated to cause the death of 2 million people per year. Physical examination, chest X-ray as well as bacterial cultures are some of the routine diagnosis procedures. A biosensor included into a microfluidic platform [107] detected samples of human and bovine tuberculosis at concentrations as low as 10 ng/mL in a 10 µL sample. The test lasted for 10 min without the need to modify the electrode surface since antibodies were attached to the surface by passive adsorption. Other biosensors for CD14 and CD16 monocyte detection as indicators of infectious state were also reported [108].

### Other bioreceptors

There are several proteins derived from antibodies such as nanobodies. Nanobodies are single-domain antibody fragments found in camelids [109], which have demonstrated to properly work as bioreceptors. A biosensor using nanobodies was constructed over a glassy carbon electrode platform, and determined the concentration of testosterone in over 1 h, achieving an LOD of 0.045 ng/mL [110]. Another case of nanobody-based impedimetric biosensor was constructed to detect rabbit IgG, whose production is very demanded for its frequent use in companies [111].

Affimers are a non-antibody scaffolds considered as a good alternative as a binding protein [44]. An impedimetric biosensor based on Affimer binding bioreceptor was used to detect and quantify Her4 protein tumour, getting an LOD lower than 1 pM in less than 30 min of sample incubation [112].

## Conclusion

In short, the evolution of impedimetric immunosensor platforms has been analysed for different types of pathogens. Parameters of strong importance such as LOD, detection time and sample volume have been discussed and compared. Alternative to classical macro-sized electrodes such as IDAM or SPE, the introduction of nanostructures such as nanoparticles or nanoporous membranes and the insertion of the biosensor into a microfluidic chip have been relevant modifications to improve these platforms. Successful evolution of biosensors is occurring and currently diagnostic techniques are becoming replaced. All biosensors described are indicated in Table 1. Nevertheless, limitations such as the high cost of the electrodes and antibodies as well as reproducibility still remain a challenge when dealing with large scale applications. As a consequence, new antibody derived proteins such as nanobodies or non-antibody proteins such as Affimers are being investigated in this field.

**Table 1:** A summarize of the different impedimetric immunosensors found in the literature

Immunosensor electrode	Analyte	Immobilisation step	LOD	Sample volume	Detection time	References
Au IDAM	*E. coli O157:H7*	MNAC/SA/BT/Ab	− 1.2 × 10^3^ cfu/mL from ground beef samples − 1.6 × 10^2^ cfu/mL from pure culture	100 μL	35 min	[54]
Au	*E. coli O157:H7*	MACA/EDC + NHS/Ab	1 × 10^3^ cfu/mL in culture	20 μL	1 h	[57]
Au	*E. coli O157:H7*	MHDA/(EDC + PFP + DIEA)/Ab/AEE	2 cfu/mL	_	45 min	[27]
Pt wire	*E. coli O157:H7 /Staphylococcus aureus*	NAM/GPMS/Ab	10^2^ cfu/mL	_	2 h	[59]
Ag/AgCl	*E. coli O157:H7*	NAM/HA/EDC + NHS/Ab	83.7 cfu/mL in milk	_	_	[61]
Au microelectrode	*E. coli O157:H7*	PANI/GLU/Ab	10^2^ cfu/mL	_	_	[62]
Au	*E. coli O157:H7*	11M1UD/ECD/HA/EDC + NHS/Ab	7 cfu/mL	1 mL	_	[2]
rGOP	*E. coli O157:H7*	Au-NPs/SA/BT/Ab/BSA	− 1.5 × 10^3^ cfu/mL cucumber − 1.5 × 10^4^ cfu/mL ground beef samples	_	_	[53]
Au	*E. coli O157:H7*	MUA/EDC + NHS/Ab/AuNPs	10^2^ cfu/mL	_	2 h	[65]
Au SPIM	*E. coli O157:H7*	DTSP/EDC + NHS/SA/BT/Ab/BSA/WGA	10^2^ cfu/mL	_	< 1 h	[66]
Au SPIM	*E. coli O157:H7*	MgNbs/SA/Biotin/Ab	1.4 × 10^3^ cfu/mL	25 μL	_	[67]
ITO	*E. coli O157:H7*	GPMS/Ab	1 cfu/mL	400 μL	45 min	[68]
Au-W microwire	*E. coli K12*	PEI/SA/BT/Ab	10^3^ cfu/mL	5 μL	_	[68]
Au	*E. coli O157:H7*	(Au-MBA-Ab), (Au-MBA-ProteinA/G-Ab), (Au-Cys-Ab), (Au-Cys-Ferrocene-Ab), (Au-Cys-PAMAM-Ferrocene-Ab)	3 cfu/mL	1 mL	90 min	[3]
Au	*S. typhimurium*	Ptyr/GLU/Ab/BSA	− 10 cfu/mL in culture − 10^2^ in milk	2 mL	3 h, 10 h respectively	[71]
Au SPE	*S. typhimurium*	Cys/Glu/Ab/BSA	− 10^3^ cfu/mL in PBS − 9 × 10^3^ in milk	1 mL	20 min	[72]
Ti-Au IDAM	*S. typhimurium*	MUA/EDC + NHS/Ab/BSA	10^3^ cfu/mL	_	30 min	[73]
Au IDAM	*S. typhimurium*	16-MHDA/SA/BT/Ab	10^2^ cfu	50 μL	1 h	[74]
Pt interdigitated microelectrodes	*S. typhi*	Au-NPs/AbPEG-thiol	10^2^ cfu/mL	10 μL	1 h	[75]
Au	SRB	MUA/EDC + NHS/lectin-ConA	1.8 cfu/mL	_	2 h	[76]
Foam Ni	SRB	AuNPs/11-MUA/EDC + NHS/Ab/BSA	2.1 × 10^1^ cfu/mL	_	2 h	[78]
Glassy carbon disc	SRB	CS + RGS/Glu/Ab/BSA	1.8 × 10^1^ cfu/mL	10 μL	1 h	[79]
Au microelectrode	*L. monocytogenes*	TiO2 nanowire/(SH-(CH2)3-CH3)/Ab	4.7 × 10^2^ cfu/mL	15 μL	50 min	[82]
IDAM	*L. monocytogenes*	MNPs/SA/BT/Ab	10^4^ cfu/mL in milk, beef and lettuce	20 nL	3 h	[83]
SPCE	*P. aeruginosa*	PP3CA/EDC + NHS/Ab	10 cfu/mL		_	[84]
Au SPE	*S. pyogenes*	Ptyr/BT/NA/BT/Ab/BSA	10^2^ cfu/ml	10 μL	30 min	[41]
Au	*S. aureus*	MHDA/EDC + NHS/Ab	10 cfu/mL	5 mL	_	[85]
Au IDAM	AI virus H5N1	Protein A/Ab/BSA	titer higher than 10^3^ EID_50_/mL	50 μL	2 h	[86]
Au IDAM	AI virus H5N1	Protein A/Ab /BSA	2^–1^ HAU/50 μL	30 μL	45 min	[38]
Au	Ad5	1,6-HDT/AuNPs/MUA/EDC + NHS/Ab	30 virus particles/mL	200 μL	_	[87]
Au	PPV	1,6-HDT/AuNPs/Ab/BSA	10 pg/mL	_	30 min	[88]
Pt wire	*Aphanomyces invadans*	G-AuNPs/SAM-Ab-BSA/GCE	309 ng/mL	_	10 min	[89]
Au SPE	TREM-1/MMP-9/HSL	Thiolated Ab	− 3.3 pM for TREM-1 − 1.1 nM for MMP-9 from mock wound fluid − 1.4 nM for HSL	10 μL	< 1 h	[52]
Au	cTnI/sLOX-1	16-MHDA/BT-caproyl-DPPE species/NA/BT/Ab	10^–13^ M each analyte in PBS and serum	_	30 min	[112]
IDAM	hTB antigen	Ab/blocking buffer	10 ng/mL	10 μL	10 min	[107]
Au	CD14/CD16 monocytes	MUA-MH/Proteing G/BSA/Ab	10^3^ cfu/ml	1 mL	2 h	[108]
Flat Au wire	Mb	MUA-MPA/EDC-NHS/Ab-Mb/BSA	5.2 ng/	_	_	[91]
Screen-printed MWCNTs	Mb	Ab-Mb/BSA	0.08 ng/mL	5 μL	_	[93]
ITO coated glass plates	Mb	APTES/EDC-NHS/Pt(MPA)/Ab-cMb/BSA	1.70 ng/mL		12 min	[94]
Au	cTnI	(MHA)/EDC-NHS/TMB/EDCH-NHS-/Dendrimer/Ab/BSA	11.7 ± 0.62 fM (0.28 ± 0.015 pg/mL)	_	1 h	[96]
Au microelectrode	D-dimer	SWCN-COOH/Ab/Casein	0.1 pg/mL (0.53fM)	_	10 min	[97]
SWCNT SPE	EGFR	CNT/EDC/NHS/Ab/BSA	2 fg/mL	_	_	[98]
Au	EGFR	AuNPS/Cys/PDITC/Proteing G/Ab	− 0.34 pg/mL in PBS − 0.88 pg/mL in human plasma	_	1 h	[99]
Au microelectrode	PSA	16MHDA/EG3SH/EDC-NHS/Amine-PEG-BT/Avidin-(BT Ab-Ag Psa-HRP Ab)	− 0.51 ng/mL for t-PSA − 0.07 ng/mL for f-PSA	_	_	[101]
Au	CA-125	MPA/EDC-NHS/AuNP@SiO2/QDs/mAb	0.0016 U/mL in serum of ovarian cancer patients	_	< 1 h	[104]
Polycrystaline Au	MDM2	Cysteamine (CA) SAM/1,4-phenylene diisothiocyanate (PDITC)/Ab/EA	0.29 pg/mL	_	_	[105]
Oxidised GCE	DHEAS	ox-GCE/AuNPs-ARG/Ab/EDC/NHS/BSA	7.4 µg/dL in blood plasma samples	_	_	[106]
GCE	Testosterone	EDC-NHS/SA/BSA/Nanobody	0.045 ng/mL	20 μL	1 h	[110]
Au	Rabbit IgG	Ptyr/Sulfo-SMCC/Nanobody	666 fM	10 μL	30 min	[111]
Au IDμE	Her4 tumour protein	Cys-Her4 Affimer/PBS-tween 20 based starting block (SB)/Her4	< 1 pM in buffer and in serum	_	30 min	[112]

Different components, steps and features of the biosensors are showed, namely: immunosensor electrode, analyte, immobilisation step, LOD and sample volume

*1,6HDT* 1,6-hexanedithiol, *11M1UD* 11-Mercapto-1-undecanol, *16MHDA* 16-Mercaptohexadecanoic, *2M2P* 2-methyl-2-propanethiol, *Ab* antibody, *AEE* 2-(2-aminoethoxy) ethanol, *APTES* 3-aminopropyltriethoxy silane, *AuNP@SiO*_*2*_ silica coated gold nanoparticles, *bTB* bovine tuberculosis, *BCNT-IL* bamboo-like multiwall carbon nanotubes-ionic liquid, *BSA* bovine serum albumina, *BT* biotin, *CA-125* cancer antigen 125, *ConA* concanavalin A, *CS* chitosan, *cTnI* Cardiac troponin I, *DHEAS* dehydroepiandrosterone sulfate, *DIEAN*,*N*-diisopropylethylamine, *DTSP* 3-dithiobis-(sulfosuccinimidyl-propionate), *EA* ethanolamine, *ECD* epichlorohydrin, *EG3SH* tri(ethylene glycol), *EGFR* epidermal growth factor, *G-AuNPs* Graphene gold nanoparticles, *GCD* glassy carbon disc, *GLU* glutaraldehyde, *GPMS* (3-glycidoxypropyl)trimethoxysilane, *Cys* cysteamine, *HA* hyaluronic acid, *hTB* human tuberculosis, *IDμE* interdigitated micro-electrode, *MACA* mercaptoacetic acid, *Mb* myoglobin, *MBA* 4-mercaptobenzoic acid, *MDM2* murine double minute 2, *MH* 6-mercapto-1-hexanol, *MHDA* mercaptohexadecanoic, *MgNbs* magnetic nanobeads, *MgNPs* magnetic nanoparticles, *MNAC* magnetic nanoparticle–antibody conjugates, *MPA* 3-mercapto propionic acid, *MUA* mercaptoundecanoic acid, *MWCNT* multi-walled carbon nanotube, *NAM* nanoporous alumina membrane, *NA* neutravidin, *PAMAM* polyamidoamine, *PANI* polyaniline, *PDITC* 1,4-phenylene diisothiocyanate, *PEG-thiol* carboxy-thiolpolyethyleneglycol, *PEI* polyethyleneimine, *PFP* 2,3,4,5,6-pentafluorophenol, *PoPD* poly (ortho-phenylenediamine; PP3CA: poly(pyrrole-3-carboxylic acid), *PSA* prostate-specific antigen, *PSSA* polystyrene sulphonic acid, *Ptyr* polytyramine, *QDs* quantum dots, *rGO* reduced graphene oxide, *rGOP* reduced graphene oxide paper, *RGS* reduced graphene sheets, *SA* streptavidin, *sLOX-1* soluble lectin-like oxidized low-density lipoprotein receptor-1, *SPCE* screen-printed carbon electrode, *SPE* screen-printed electrode, *SPIE* screen-printed interdigitated electrode, *Sulfo-SMCC* sulfosuccinimidyl 4-[*N*-maleimidomethyl] cyclohexane-1-carboxylate, *SWCN* single-walled carbon nanotube, *TMB* 3,3′,5,5′-tetramethyl benzidine, *VACNT* vertically aligned carbon nanotube, *WGA* wheat germ agglutinin
